# The ambiguous role of obesity in oncology by promoting cancer but boosting antitumor immunotherapy

**DOI:** 10.1186/s12929-022-00796-0

**Published:** 2022-02-14

**Authors:** José Antônio Fagundes Assumpção, Gabriel Pasquarelli-do-Nascimento, Mariana Saldanha Viegas Duarte, Martín Hernan Bonamino, Kelly Grace Magalhães

**Affiliations:** 1 Laboratory of Immunology and Inflammation, Department of Cell Biology, University of Brasilia, Brasília, DF Brazil; 2 Immunology and Tumor Biology Program - Research Coordination, Brazilian National Cancer Institute (INCA), Rio de Janeiro, Brazil; 3 Vice - Presidency of Research and Biological Collections (VPPCB), Oswaldo Cruz Foundation (FIOCRUZ), Rio de Janeiro, Brazil

**Keywords:** Obesity, Cancer, Immunotherapy, Adipose tissue, Inflammation

## Abstract

Obesity is nowadays considered a pandemic which prevalence’s has been steadily increasingly in western countries. It is a dynamic, complex, and multifactorial disease which propitiates the development of several metabolic and cardiovascular diseases, as well as cancer. Excessive adipose tissue has been causally related to cancer progression and is a preventable risk factor for overall and cancer-specific survival, associated with poor prognosis in cancer patients. The onset of obesity features a state of chronic low-grade inflammation and secretion of a diversity of adipocyte-derived molecules (adipokines, cytokines, hormones), responsible for altering the metabolic, inflammatory, and immune landscape. The crosstalk between adipocytes and tumor cells fuels the tumor microenvironment with pro-inflammatory factors, promoting tissue injury, mutagenesis, invasion, and metastasis. Although classically established as a risk factor for cancer and treatment toxicity, recent evidence suggests mild obesity is related to better outcomes, with obese cancer patients showing better responses to treatment when compared to lean cancer patients. This phenomenon is termed obesity paradox and has been reported in different types and stages of cancer. The mechanisms underlying this paradoxical relationship between obesity and cancer are still not fully described but point to systemic alterations in metabolic fitness and modulation of the tumor microenvironment by obesity-associated molecules. Obesity impacts the response to cancer treatments, such as chemotherapy and immunotherapy, and has been reported as having a positive association with immune checkpoint therapy. In this review, we discuss obesity’s association to inflammation and cancer, also highlighting potential physiological and biological mechanisms underlying this association, hoping to clarify the existence and impact of obesity paradox in cancer development and treatment.

## Introduction

Although extensively researched, obesity and cancer are both pathologies exhibiting steadily-increasing and fast-paced expansion worldwide. In 2015, 2 billion people (27% of the world population) was considered overweight [1]. Alongside, cancer incidence also increased in recent years. In 2018, there were 18.1 million new cancer cases worldwide [2]. These ongoing epidemics accounted for over 4 million and 9.6 million deaths, respectively, amounting for billions of dollars in costs related to treatment and hospitalization [3–5]. This great toll presents major challenges to healthcare systems worldwide. Western countries, in particular, show increasing prevalence of obesity and obesity-related morbidities [6]. Regarding cancer, a similar situation can be described. Although some types of cancer showed a decrease in death rates in the last 20 years—due to better understanding and treatment of the disease, cancer cases are on the rise [2, 7, 8]. A 60% increase in the number of cancer cases is expected in the next 20 years [9]. In low- and middle-income countries, this increase could reach 81%, as developmental and economic factors also influence the expansion of both diseases [10–13].

The onset of obesity is linked to increased occurrence of cancer (one of many obesity-associated diseases) and a more aggressive, metastatic phenotype of tumor cells [14, 15]. This link is usually defined in terms of the systemic low-grade chronic obesity-induced inflammation, considered a hallmark of cancer establishment and progression [16, 17]. Changes in adipose tissue composition, function, and secretion have a direct impact on tumor microenvironment (TME) and the immune system [18, 19]. Both weight loss and the transdifferentiation between different types of adipose tissue (white, brown, and beige) are considered viable strategies to prevent cancer incidence. While the white adipose tissue (WAT) secretes a plethora of inflammatory mediators (adipokines, cytokines, hormones), inducing tissue damage/remodeling and propitiating cancer progression, the beige and brown adipose tissues (BAT) are responsible for adaptive energy expenditure, inducing thermogenesis and protecting against obesity and, therefore, obesity-related cancer [20, 21]. The functioning of cancer cells and the organization of the TME are affected not only by adipocyte dysfunction but also by immune cells residing in the adipose tissue microenvironment (ATM), such as macrophages and lymphocytes [22].

Although obesity is classically considered a pro-carcinogenic condition, in some cases, overweight and/or obesity seemingly induces a protective status against certain stages and types of cancer, since it may boost anti-tumor immunotherapy. This conflictual idea is termed obesity paradox and entails improved outcomes of overweight and class 1 obesity (Body Mass Index-BMI = 25–34.9 kg/m^2^) cancer patients when compared to lean, although this effect is not observed in all patients and cancers [23, 24]. As for the reasons why this takes place, a few hypotheses, both methodological and epidemiological, come to mind, such as biases in the use of BMI as a reliable measure of obesity and body composition, the use of excessive adipose tissue as an energy reserve (improving response to treatment), and better absorption, metabolism, and response to treatment in obese patients [25–27]. Also, loss of adipose and muscle tissue (cachexia and sarcopenia) is associated with higher toxicity and mortality of cancer patients, suggesting mild obesity could compensate for this loss, actually turning the accumulation of adipose tissue into a protective tool against cancer development and/or progression [28, 29]. It is important to highlight obesity is still considered a risk factor for most types of cancer, even non-obesity-associated tumors and tumors in which prevalence is not related to obesity. Severe obesity does not confer the same advantages, which raises questions about which mechanisms take part pro- and anti-tumoral properties of adipose tissue.

The mechanisms and pathways ruling this co-regulation between adipose tissue and the TME are yet to be fully described, but evidence points to the involvement of the immune system’s inflammatory and cellular response, through mediators exchanged between the adipose tissue, immune cells, and cancer cells [30]. Considering the variety of adipocytes, cancer cells and immune cells in the TME, tumorigenesis and tumor progression are targeted by cells (or secretion from these cells) with diverse functioning and morphology, acting antagonistically or synergically, depending on a wide range of factors (number, activation, polarization, localization) [31, 32].

Given the metabolic, inflammatory, and immune abnormalities of obese cancer patients, together with the complex crosstalk between adipocytes and cancer cells, and its implications in cancer treatment, this review focuses on debating the implications of obesity and the obesity paradox in cancer development and immunotherapy response, as well as diving into the physiological and biological mechanisms underlying this paradox.

## Obesity and inflammation modulation

### Obesity and metabolic syndrome: global health burdens

A direct consequence of lifestyle changes, the statistics regarding obesity are escalating worldwide, impacting more than 6 hundred thousand people in 2016. Although the definition and classification of obesity are controversial, patients displaying a BMI equal or higher than 30 kg/m^2^ are considered as presenting the obese phenotype (WHO, 2020). Obesity is intimately related to elevated risk of developing a large set of pathologies as it associates with chronic low-grade inflammation in many tissues, as adipose tissue (AT), liver, skeletal muscle, intestine, pancreatic islets, and brain [33–36]. Its inflammatory characteristics cope obesity with type 2 diabetes (T2D), heart disorders, non-alcoholic fatty liver diseases (NAFLDs), and certain malignancies, conditions which elevated morbidity favor the increased death rate characteristic of the obese state [37, 38].

The most frequent component of metabolic syndrome (MS) [39], abdominal obesity is characterized to be a waist circumference of more than 102 cm in men and more than 88 cm in women [40]. Elevated amount of visceral AT (VAT) relates to increased soluble tumor necrosis factor-alpha (TNF-α) receptor 2 [41], incident hypertension [42], and adverse metabolic risk profile [43]. Although the criteria for defining and diagnosing MS has changed throughout the years [44], this lifestyle disease drastically increases the risk of cardiovascular and cerebrovascular disorders [45] and associates with insulin resistance (IR) [46].

### Subcellular disturbances and oxidative stress

Several studies connect adipocyte subcellular alterations with the inflammatory and metabolic derangements observed in MS-affected individuals, including disrupted mitochondrial and endoplasmic reticulum (ER) functions [47, 48]. Excessive food intake favors reactive oxygen species (ROS) production and mitochondrial dynamic dysfunction [49]. Mitochondria, the powerhouse of the cell, oxidize glucose and free fatty acids (FFAs) to produce adenosine triphosphate (ATP) [50]. However, this small organelle is a relevant ROS source inside the cell [51]: nutrient excess in adipocytes leads to augmented electron supply to the mitochondria electron transport chain (ETC) [52], favoring ROS generation. ROS damages mitochondrial constituents [53], impacting on cell viability [54]. Mitochondrial dysfunction contribute to VAT inflammation through the ROS and mitochondrial-derived damage-associated molecular patterns (DAMPs) release [55].

Nutrient overload is also implicated in endoplasmic reticulum (ER) stress [56]. The largest organelle in the cell, ER can be found at the cell endoplasm [57] and is the main actor in protein production, calcium (Ca2+) storage, and lipid biosynthesis [58]. When proteins accumulate in ER lumen, the homeostatic mechanism unfolded protein response (UPR) is initiated [59], leading to ROS formation and ER stress [60]. ER stress influence VAT inflammatory status through inducing TNFα and IL-6 expression, activating JNK pathway [61], and favoring nuclear factor kappa-light chain-enhancer of activated B cells (NF-κB) signaling [62].

### Adipocyte cell death and AT inflammation

An evidence that diets rich in saturated lipids directly influence MS onset is that progressive lipid accumulation in adipocytes correlates with cell hypertrophy and enlarged VAT, which cause adipocyte stress and cytokine secretion [63]. The inflammatory and oxidative microenvironment of VAT impairs healthy adipose expansion [64]. Although adipocytes secrete several inflammatory molecules [65, 66], most mediators are produced by immune cells present in VAT of obese animals and humans [34]. During metabolic syndrome, these inflammatory cells secrete massive amounts of proinflammatory cytokines, as Interferon γ (IFNγ), Interleukin 6 (IL-6), and TNFα [35, 67, 68], lipid mediators, as Leukotriene B_4_ (LTB_4_) [69], and chemokines, as Monocyte Chemoattractant Protein-1 (MCP1) [70–72]. Stressed adipocytes present deregulated adipokine secretory pattern, secreting more proinflammatory adipokines, as leptin, and less of the anti-inflammatory adiponectin [73]. The key players in VAT chronic inflammation are decreased Th2 cells [74] and Treg [75] and increased neutrophils [76], mast cells [77], innate lymphoid cells (ILCs) [78, 79], CD8- [80] and CD4-positive T cells (Th1 and Th17) [81], B cells (B2) [82], and macrophages (M1-like) [83, 84].

T and B cells and macrophages form crown-like structures (CLSs) around dead or dying adipocytes [80, 85, 86], which display apoptotic and pyroptotic cell death signaling [87, 88]. Differently from apoptosis [89], pyroptosis depends on inflammasome activity for membrane pore formation and secretion of IL-18 and IL-1β [90, 91]. NLR family pyrin domain containing 3 (NLRP3) and absent in melanoma 2 (AIM2) inflammasomes are molecular pattern sensors [92] that, through processing caspase-1, enable cytokine secretion and maturation of Gasdermin D (GSDMD), the cell death executor [90]. Membrane integrity loss leads to extravasation of intracellular contents including HMGB1 [93, 94], an alarming that, in conjunction with other intracellular factors, as ATP [95], amplifies VAT inflammation [88].

The rapid VAT enlargement and the increased VAT oxygen consumption that cause MS favors the occurrence of hypoxia, which enhance adipocyte MCP-1 and LTB_4_ secretion [96] and increase macrophage M1-like polarization [97]. A MS component [98], elevated fasting glucose leads to enhanced advanced glycation end products (AGEs) and its receptor (RAGE), which induce AIM2 inflammasome activation [99, 100]. In addition, persistent hyperglycemia in rodent models modulates gut immune function and microbiota [101]. Also highlighting the impact of diet on MS development, high-fat diet (HFD) mouse models display exacerbated VAT inflammation [102] due to the action of saturated FFAs on NLRP3 [103], and Toll-like receptors-2 (TLR-2) and 4 (TLR-4) [104]. Microbiota is also altered by HFD, favoring increased lipopolysaccharide (LPS)-containing bacteria and augmented intestinal permeability [105], resulting in metabolic endotoxemia and systemic inflammation worsening [106], implicating dysbiosis in MS pathology [107].

### AT inflammation and insulin resistance

IR strongly correlates with MS [108]. IR was first observed by Himsworth et al., who could verify that individuals display discrepant blood glycemia after insulin infusion [109]. Many studies connect IR with oxidative stress and chronic low-grade inflammation [110, 111]. Increased VAT levels of proinflammatory mediators, including MCP-1 [112], IFN γ [80, 113], TNF [65, 83], and IL-6 [114] induce insulin resistance (IR) in VAT. In addition, studies evaluating the absence of neutrophils [76], mast cells [77], ILCs [79, 115], CD8- [80] and CD4-positive T cells (Th1 and Th17) [113], B cells (B2) [82], and macrophages (M1-like) [65, 112] inform improved VAT IR. M1-like migration to VAT was shown to precede IR in HFD mouse model [116]. The anti-inflammatory adiponectin, downregulated in subjects with obesity and T2D [117], is also an insulin sensitizer [118]. In addition to the effect of caspase-1 on IR through modulating inflammatory status, this protein also influences VAT metabolic function [119]. Caspase-1 and inflammasome activity influence the gut microbiota as well [120], and human and mouse experimental endotoxemia studies indicate circulating LPS as an IR inducer [121].

Inflammation exacerbate IR and vice versa [36, 122]. IR is associated with many detrimental systemic effects [122]. As IR impair proper glucose uptake by skeletal muscle and favor liver gluconeogenesis, hyperglycemia leads to pancreatic cytotoxicity and β-cell death [36, 123]. Furthermore, IR induces VAT hormone sensitive lipase activity and lipolysis, which increase the levels of circulating FFA [37], and the effects of inflammation on preadipocyte/adipocyte favor ectopic fat deposition in organs as liver and skeletal muscle, leading to systemic IR [36, 123]. Considering their detrimental effects on VAT physiology here described, oxidative stress, chronic low-grade inflammation, and IR are considered to drive metabolic syndrome, enhancing the risk for several life-threatening diseases [45].

**Fig. 1:**
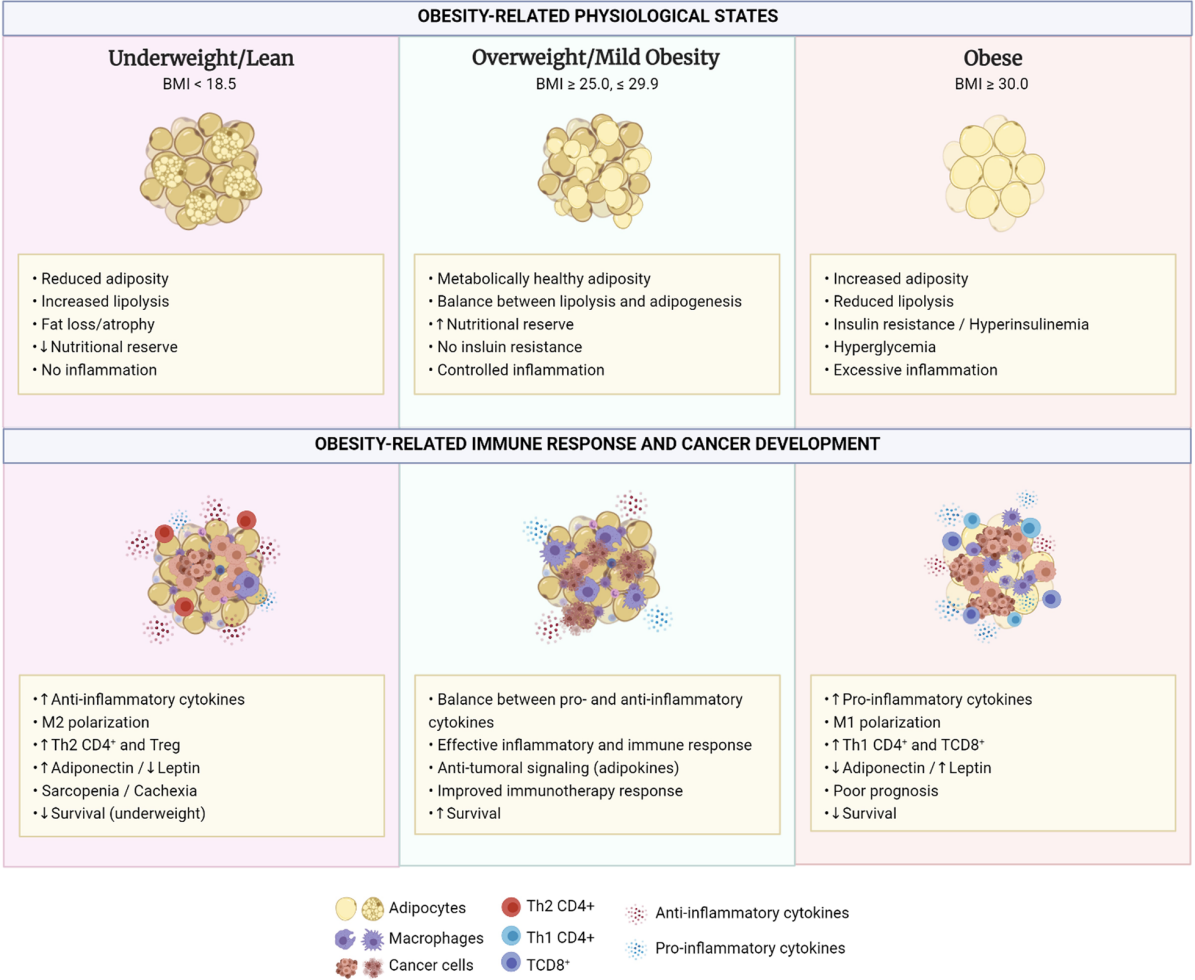
Obesity-related physiological states, immune response and cancer development. Comparison between underweight/lean, overweight/mild obesity and obese phenotypes, regarding their physiological states, immune responses and cancer development. While both extremes—underweight and obese—show poor survival and disadvantages regarding cancer development and progression, mild obesity, in overweight patients, has been described as a protective phenotype, allowing for improved immunotherapy response and survival of cancer patients

Therefore, exacerbated food intake and sedentary lifestyle leads to mitochondrial dysfunction and ER stress, subcellular disturbances connected to VAT oxidative stress and inflammation. The disrupted inflammatory status in VAT favors immune cells infiltration, proliferation, and polarization to proinflammatory profiles. VAT dysfunction and the stressful microenvironment induce adipocyte apoptotic and pyroptotic cell death, which further exacerbate inflammation. Dietary factors influence systemic and VAT inflammatory status through impacting on immune cells and on microbiota, the latter being key on regulating intestinal barrier integrity and, during dysbiosis, cope with elevated circulating LPS level (Fig. 1). Inflammatory status is intimately related to insulin sensitivity through several molecular pathways, and IR and inflammation, exacerbating each other, act as drivers of the elevated morbidity, mortality, and financial burden of metabolic syndrome.

## Obesity, cancer, and the obesity paradox

### Cancer: definition and pathology statistics

Cancer, rather than a single disease, is a set of over 100 pathologies which share a common denominator: excessive proliferation. The transformation of normal cells into malignant cells is a largely studied, but still not fully understood process with multiple causes, a different perspective of the previous genetic-driven transformation [124]. The recently established—and continuously updated—hallmarks of cancer, comprise multiple characteristics and capacities which, when acquired or expressed by cells, allow for sustained proliferation, resistance to cell death, immortality, and enable tumoral cells to grow and spread throughout the organism, ultimately enabling the emergence of malignant tumors, i.e., cancer [125].

In 2020, in the United States alone, 1,806,590 diagnosed cancer cases are estimated (around 4950 new cases/day). Prostate, breast, lung, colon, and skin make up roughly half of the sites of tumor development. Regarding cancer-related deaths, it is estimated that 606,520 individuals perished from cancer in 2020. Even though in the last 20 years or so the cancer incidence rate for men and women has stabilized (due to better understanding, diagnosis, and screening), cancer is still the second leading cause of deaths in the United States. Risk factors for the development of cancer include not only genetic predisposition, but also the exposure to a gamut of environmental and behavioral factors, such as excessive alcohol consumption, cigarette smoking, infections, and, as discussed here, obesity [126, 127].

### Obesity and cancer: overview of a complex relationship

According to data from the American Cancer Society, in 2014, 7.8% (122,536) of all cancers in the United States and 6.5% (38,188)  of all cancer deaths were attributed to overweight or obesity [128]. Globally, estimations point to 481,000 new cancer cases being related to obesity, designating excessive body adiposity as a well-established risk factor for cancer development [129]. These cases, however, are not equally distributed between all cancer types and countries. In men, two thirds of these cases were from kidney and colon cancer, while, in women, approximately three quarters were from postmenopausal breast, corpus uteri and colon cancer [130, 131]. As Whiteman and Wilson pointed, the United States had the highest fraction of colorectal, pancreatic, and postmenopausal breast cancer cases attributable to overweight and obesity [132]. Added to the fact that, also in the United States, there has been a fast-paced growth in overweight and obesity rates, with 66% of adults and 33% of children being considered overweight or obese, the landscape is nothing short of alarming [133]. This trend seems to also be true to developing countries, such as Brazil, where the aforementioned cancers account for just about half of all cancer cases identified in the country by the year 2012 [134].

**Fig. 2:**
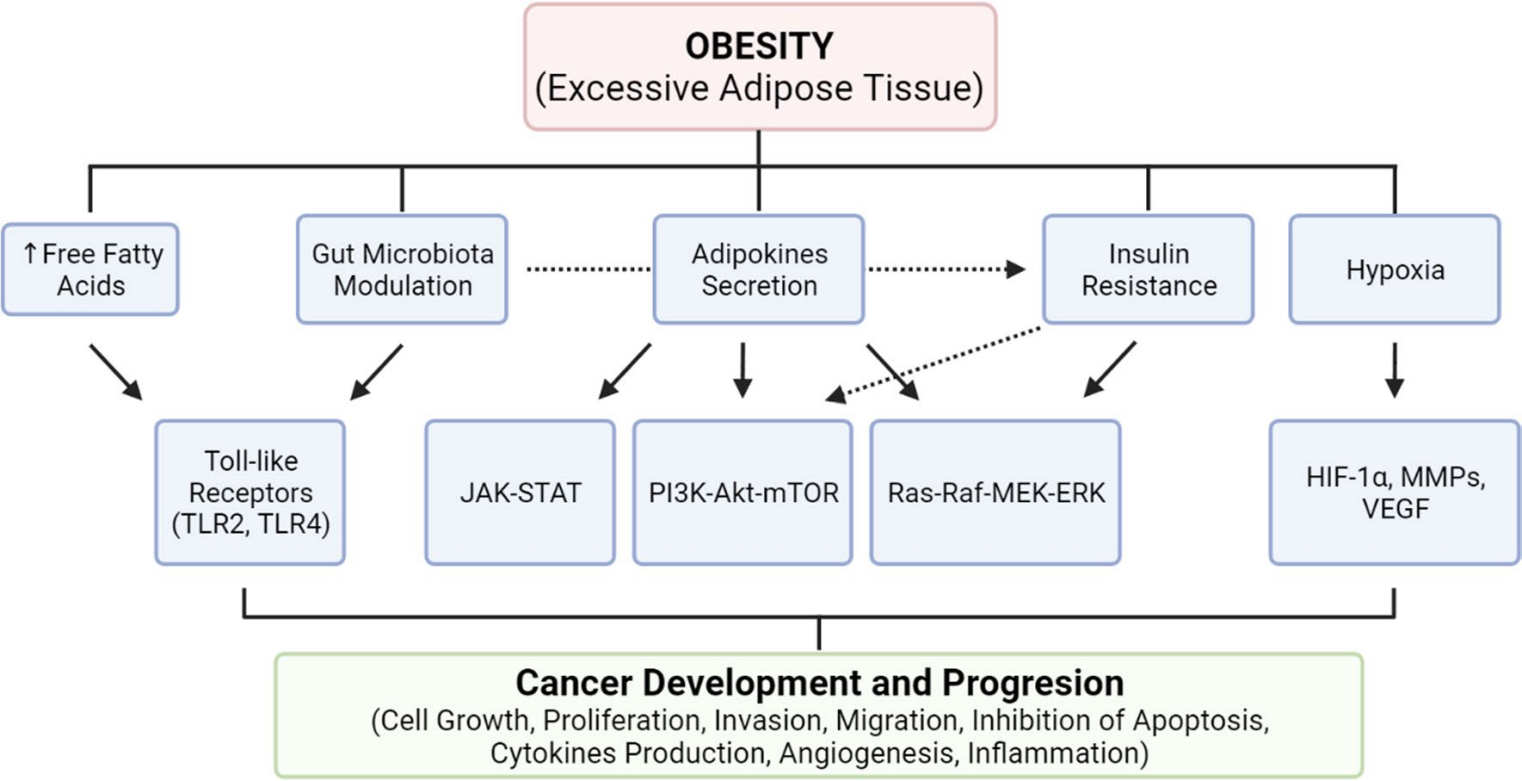
Biological mechanisms of obesity in cancer development and progression. Pathophysiological mechanisms regarding obesity effects upon cancer development and progression. The secretion of adipokines and release of free fatty acids by adipocytes induces several different systemic and cellular responses. The combination of metabolic alterations induced by the adipose tissue results in the activation of several signaling pathways responsible for cellular growth, proliferation, invasion, migration, inhibition of apoptosis, angiogenesis and induction of inflammation, all of which are hallmarks of cancer development

A variety of different types of cancer have already been described as having an increased risk when associated to excessive body fatness, such as cancers from the gastrointestinal tract (esophagus, cardia, stomach, pancreas, gallbladder, liver, colon, rectum), breast (postmenopausal, specifically ER+ breast cancer risk, whereas ER− or TNBC is little or inversely associated with obesity), kidney, thyroid, prostate, ovary, endometrium, multiple myeloma, and meningioma [135, 136]. There is significant evidence of positive associations between obesity and cancer [129]. Excessive body fatness is tied to systemic and tumor microenvironmental inflammation, usually reported as a chronic low-grade pro-inflammatory state, altering the immune response, insulin resistance, insulin-like growth factors, and sex hormones pathways, inducing specific lipids, and secreting various adipokines and inflammatory cytokines [23, 137–139]. In league, these obesity-related processes account for a thriving environment for tumor initiation, development, and progression [136, 140, 141] (Fig. 2).

Seemingly paradoxical reports for other cancers, however, indicate that overweight (BMI ≥ 25 kg/m^2^ and obesity (BMI ≥ 30 kg/m^2^) are related to more favorable long-term survival post-surgery or treatment. Patients showed increased long-term, recurrence-free, and overall survival, while displaying reduced morbidities in different cancer types, such as renal cell carcinoma and non-small cell lung cancer (NSCLC), suggesting high BMI as an independent predictor for better cancer survival [28, 142–145].

### The obesity paradox

In face of such opposing reports, studies termed the occurrence of the obesity paradox, a different relation between obesity as a risk factor for diseases, including cancer, in which higher adiposity (primarily determined using the BMI index) is associated to better survival, response to treatment, and better clinical outcomes, different from the commonplace where a higher BMI index would aid disease development or point to reduced chances of survival [28, 146].

The obesity paradox was first described in diseases and conditions other than cancer, with supporting literature regarding cardiovascular disease (CVD), atherosclerosis, kidney failure, and T2D [147–151]. Review of clinical and epidemiological literature shows some types of cancer also fit the obesity paradox. In patients with lung, bladder, breast cancer (premenopausal), and melanoma, to name a few, overweight/obese patients displayed better long-term survival rate, reduced overall morbidity, and reduced in-hospital mortality [142, 152].

These studies endorse the complexity of obesity and its effects upon the metabolism, considering that, for some types of cancer, the obese phenotype predicts for better clinical outcomes and long-term prognosis. Zhang et al. (2017) regarded the obesity paradox as an under-explored scientific fact, rather than an unexpected paradox—a point of view shared by this review. The collective evidence supports both a deleterious and a protective role for obesity in cancer, reflected in its definition as a risk factor for cancer development and as a protective phenotype for established cancers. A common factor in most conditions in which the obesity paradox is observed is the perpetuation of inflammation. The lack of inflammation resolution is central to the development of chronic diseases and tumoral growth and expansion [147]. By fostering the development of cancer, inflammation and immune mediators may be pieces of the puzzle for explaining the cellular and molecular basis of the obesity paradox. Even so, this line of research is still in need of further exploration [125, 147, 153].

There is a lot of debate about the obesity paradox and potential issues with previous studies in which it was described. Namely, aspects such as confounding variables (smoking, age and other comorbidities), reverse causation (weight change as a consequence rather than a cause of cancer), differences in attentive and medical care (control of hypertension and prevention of hyperglycemia in obese patients), collider stratification biases and the fact that BMI (the most preponderant measure for obesity) does not differentiate between subcutaneous (SAT), visceral (VAT), and other types of adipose tissue [23, 28, 136, 142, 154]. When examining previous meta-analyses, attention is also given for the timing of BMI measurement, if pre- or post-diagnosis, treatment, or surgery [136, 155].These are usually considered limitations in research concerning the obesity paradox, for they may partly explain the observed differences in obesity’s effects upon each type of cancer. Since many extensive reviews take part on describing the aspects for why this paradox may or may not be true, other than an illusionary causality [23, 136], we focus on the physiological states and biological mechanisms which possibly underlie these observations, also commenting upon the impacts of adiposity over the immune system and immunotherapy, in an effort to provide biological basis for the results and evidence present in the literature.

## Obesity and cancer-related physiological states

### Healthy versus unhealthy obesity

Obesity is recognized as an adverse factor in the outset of a wide gamut of diseases, metabolic disorders, and several types of cancer [156]. It is the main cause of CVD, cancer mortality and all-cause mortality [157–160]. Notwithstanding, a proportion of obese individuals might benefit from the obesity-related phenotype and show decreased risk for obesity-related metabolic complications, despite having a high BMI [161, 162]. This has been credited to differences in body fat distribution and heterogeneous cardiometabolic profiles, originating the ‘metabolically healthy obese’ (MHO) subgroup [157, 163], comprising obese individuals (BMI > 30 kg/m^2^) with reduced risk of cardiovascular morbidity and mortality [156]. This subgroup is classified as obese, even though individuals do not have MS and show appropriate metabolic profile [157]. Populational frequencies range from 10 to 50%, with recent reports of an overall prevalence of 35% among 40 populations of obese adults [164, 165].

Studies comparing different obese subgroups and considering the stratification by presence or absence of MS show distinct rates for total mortality [166, 167]. In general, MHO individuals have lower risk of cardiovascular and all-cause mortality when compared to metabolically unhealthy obese (MUO) and metabolically unhealthy non-obese (MUNO) individuals [168]. In certain populations, these differences are also observed when comparing metabolically healthy obese (MHO) to metabolically healthy non-obese (MHNO) individuals, indicating metabolic capability has an overall stronger impact upon mortality than BMI alone, since poor metabolic health status contributes more to mortality than high BMI indexes [169, 170]. Besides adipose tissue distribution and insulin resistance, metabolic risk factors encompass lipid profiling, blood pressure, inflammation, and physical fitness [156].

When discussing the physiological foundation and biological mechanisms ruling metabolic health obesity—as well as cancer development-, genetically modified mouse models of metabolically healthy obesity are a useful tool. Leptin-deficient mice, or those overexpressing the mitochondrial outer membrane protein (mitoNEET), or adipose-specific glucose transporter type 4, show increased non-visceral adipose tissue deposition, expansion of subcutaneous adipose tissue, reduced fat content in the liver and a metabolically beneficial adipocytokine secretion pattern [171, 172]. Immune-wise, these mice exhibit reduced infiltration of macrophages in adipose tissue, and low systemic inflammation. A plausible explanation is that higher subcutaneous adipose content, supported by adipose tissue de novo lipogenesis, in conjunction with reduced inflammatory signaling, enable a healthier phenotype [173–176]. Concurrently, in humans, observations about the MHO phenotype are similar to those of animal models. Metabolic healthy obese individuals present reduced fat depots in the skeletal muscle and liver fat deposition (related to insulin sensitivity), while also displaying expansion of subcutaneous adipose tissue [177, 178].

Together, improved metabolic health and better control over inflammation, MHO patients (despite a higher BMI), show better prognosis against cancer development and cancer-related adversities even when compared to lean (metabolically unhealthy) patients. In conjunction, they promote tolerance to cancer therapy and post-surgery complications, resulting in improved long-term survival [24, 28]. Previous studies shed a light on the incidence rates of gastric cancer in MHO patients. Compared to all other subtypes, the incidence and hazard ratios of gastric cancer were lower than all other groups [179]. Similarly, the risks of colorectal neoplasia in MHO individuals are lower when compared to their non-obese counterparts, whether healthy or unhealthy [180]. The same is true to bladder cancer [181].

When MHO subjects are compared to lean controls, this advantage may not always be observed, or even be inversed back to the usual obesity-as-a-risk-factor scenario. A 20 year follow-up showed no difference in mortality and CV morbidity risks when comparing MHO to non-obese subjects [182]. When discussing cancer—or the obesity paradox, for that matter, it is not unusual for studies to contradict each other. Not all cancer types behave similarly, and confirming this, the MHO phenotype was linked to increased incidence of gastroesophageal reflux disease and erosive esophagitis (risk factors for esophageal adenocarcinoma) when compared to other metabolically healthy groups [183, 184]. A large scale (12,542,390 participants) comparison between MHO and MHNO individuals for incidence of any type of cancer showed obesity’s effects outrange metabolic health and increase the risk of developing cancer [185].

In the face of the divergent studies about metabolically health obesity, and its fairly recent definition, there are limitations for considering it an advantage, at least concerning cancer development and pathology. Not only metabolic health status is a transient phenotype, but there is a lack of uniformity in the outcome of studies concerning cancer and a few other diseases [180–183, 186]. Obesity is not, in most situations, a safe condition which would justify lack of treatment or consideration. Even without metabolic dysfunction, therapeutic weight loss is still instructed to obese individuals [185]. Nevertheless, metabolically health obesity can serve as a model for mechanistic studies regarding obesity and obesity-associated diseases. Also, in the clinical scenario, it might assist in a more personalized and risk-stratified obesity treatment, since obesity is taken into consideration when defining therapeutic procedures and predicting patient’s prognosis of cancer [28, 187].

### Meet me halfway: too lean is also not good

Another physiological state that needs to be thoroughly considered in the context of cancer and body weight, is cachexia. Cachexia’s exact definition is still debated, but it is regarded as a metabolic syndrome associated to disease, a systemic inflammatory response marked by loss of muscle mass (sarcopenia), associated or not to loss of fat mass, resulting in functional decline because of a negative protein-energy balance [188, 189]. It is often cited as a paraneoplastic syndrome, i.e., symptoms resulting from tissue damage outside the tumor site, due to the crosstalk between the tumor and the immune system [190]. Cachexia is related to 20% of cancer-related deaths and displays high prevalence in pancreatic and gastric cancer (87%), as well as colon, lung, prostate (61%) and breast (40%) cancer. Expectedly, it is an indicator of poor prognosis and increased mortality in cancer patients [191–193]. The unbalance in nutritional homeostasis is mediated by tumor-induced alterations in the metabolism, impairing the capacity to drive-off infections and resist the adverse effects of chemotherapy and radiotherapy. Cachexia also reduces the efficacy of anti-cancer treatments [191, 194]. Besides cancer, cachexia is also associated with an array of acute and chronic inflammatory conditions [195–197].

In the context of obesity, obesity-associated cancer, and the obesity paradox, overweight and obese subjects would have larger nutritional reserves than lean subjects, better dealing with the adverse effects of cachexia and better resisting surgical interventions [142]. This implies that patients with higher BMI are less vulnerable to cachexia-induced effects, displaying a more favorable outcome [28]. Considering cachexia and sarcopenia account for reductions in BMI, having a higher BMI indicates a tendency for better prognosis, as described in studies comparing obese, underweight, and lean patients with lung cancer and metastatic melanoma [193, 198, 199]. However, there is a catch. Obesity is very heterogeneous, and the ratio between fat and muscle mass is highly variable between individuals [200]. A know phenomenon which can be overlooked in patients with high BMI is sarcopenic obesity, in which excessive adiposity is accompanied by sarcopenia [201, 202]. Indeed, computed tomography (CT) scans reveal that the conventional criteria of assessing obesity using BMI is not precise enough for the detection of cachexia and sarcopenia, regardless of overall body weight [203]. In patients with breast cancer, postdiagnosis weight gain is often associated with loss of muscle mass, rendering BMI alone as an ineffective tool to assess sarcopenic obesity [204].

Accumulated evidence shows sarcopenic obesity as a predictor of morbidity and mortality in cancer, emphasizing the importance of lean mass maintenance in survival. Cachexia and sarcopenia are related to poor outcome and increased chemotherapy-induced toxicity in patients with lung cancer. In pancreatic cancer, they predict survival and worst postoperative outcome. In solid tumors from the respiratory and gastrointestinal tract, cachexia indicates poorer functional status [189, 205, 206].

Another important hallmark of cancer cachexia is systemic inflammation and an increase in pro-inflammatory cytokines, fueling cancer progression [188, 207, 208]. In cachexia, WAT persistently produces and secretes pro-inflammatory mediators, contributing to the onset and persistence of inflammation [209, 210]. Increased expression of TNF-α, IL-1β and IL-10 was observed in WAT adipocytes from different regions [211]. During cancer-associated cachexia, SAT could impact early cancer development, secreting higher numbers of inflammatory cytokines when compared to the mesenteric adipose tissue [212]. Regulation of inflammation in the adipocytes is carried on by NF‐κB and inflammasome pathways activation [209, 211]. Indeed, in cachectic cancer patients, there is increased expression of NF-κBp65 and its target genes [209]. NLRP3 inflammasome and caspase-1 pathways are also activated, given the secretion of IL-1β [213–215]. Knockout animal models for NLRP3 and caspase-1 are resistant to the development of obesity and show enhanced insulin sensitivity when compared to controls [211, 216, 217].

Cancer and cachexia are also accompanied of adipose atrophy (mainly loss of WAT), through increased adipocyte lipolysis, reduced lipid deposition, and decreased adipogenesis. In cancer patients, these factors have been linked to reduced quality of life and shorter survival, regardless of BMI [28, 218]. Remarkably, in animal models of cancer cachectic mice, browning of WAT precedes skeletal muscle atrophy [219]. It is suggested that inhibiting the switch from white to brown adipose tissue prevents severe loss of muscle mass and that alterations in the adipose tissue metabolism are a prerequisite for skeletal muscle atrophy [220, 221]. Indeed, blocking adipocyte lipolysis and browning seems to protect mice from lung carcinoma-induced cachexia, improving survival [222, 223]. The exact mechanisms that mediate the crosstalk between loss of fat mas to skeletal muscle atrophy are still not clear [224]. It has been proposed that inflammation, induction of lipases and reduced AMP-activated protein kinase activity contribute to tumor-induced lipolysis [225, 226]. By storing a larger quantity of adipose tissue, obese patients could have an advantage when dealing with the metabolic demand and wasting conditions of cancer and cachexia, preventing both adipose and muscular tissue loss, or at least better resisting these processes, which makes sense partly explaining the protective role of excessive adiposity (i.e., obesity paradox) in cancer and cachexia [28, 227].

### Home is where good fat is

When diagnosing adiposity, BMI is still predominant, disregarding its inability to differentiate lean mass from adipose tissue, and/or the different types and locations of adipose tissue, which possess distinguishable functioning and reverberations upon metabolism and cancer progression [228]. Accordingly, obesity-induced metabolic abnormalities are often associated to adipose tissue location, rather than total amount of adipose tissue [229, 230]. In obesity, VAT and SAT, which have distinct morphology and function, are of major interest [231, 232]. 5–20% of total body fat is constituted of VAT, while SAT accounts for approximately 80% [233]. SAT is often described as benign (or mildly related to disease development), insulin-sensitive, less lipolytic and more lipogenic, while VAT, is related to elevated lipid turnover, reduced plasticity (transdifferentiation), higher vascularization, and is usually infiltrated with immune cells (macrophages and lymphocytes) [232, 234, 235].

VAT is considered an endocrine organ, responsible for synthesizing several molecules which regulate appetite, innate and adaptive immunity, hematopoiesis, and angiogenesis, i.e., processes involved in physiologic and homeostatic maintenance, but also pathologic development. VAT is a known causal factor of insulin resistance, hypertension, CVD, and cancer [98, 236–238]. VAT secretes proinflammatory mediators, growth factors, hormones (such as estrogen), and adipokines, all of which contribute to the establishment and progression of diseases [237, 239]. Up to now, over 15 adipokines have been related to cancer progression, a number which is expected to grow with the advance of research in this area [240, 241].

Synergistically, FFAs resulting from VAT lipolysis act upon the liver, inducing insulin resistance and altering glucose metabolism. FFAs stimulate mutagenic pathways and contribute to proliferation, growth, and migration of cancer cells [231, 233]. FFAs modulate gene expression, activate the mTOR/PI3K pathway, reduce the expression of inhibitors of cell proliferation and promote metastasis [242–244]. The proximity of VAT to vital organs (liver, heart, and colon) contributes to its role in inducing pathophysiological alterations such as metabolic syndrome and cancer [245, 246]. In contrast, large depots of SAT are associated with better prognosis, reduced mortality risk and better overall survival in gastrointestinal, respiratory, kidney and prostate cancer [247, 248].

Considering the extensive distinction between subsets of adipose tissue, in the clinic, it would be recommended to measure body fat and adipose tissue distribution using more accurate parameters, other than BMI. Waist circumference, waist-to-hip ratio, and skinfold thickness are more strongly associated with visceral fat and cancer risk than BMI, even though evidence is conflicting, since these methods define both VAT and SAT at the waist level [138, 153, 249]. Other techniques, such as dual-energy X-ray absorptiometry, bioelectrical impedance analysis, and computed tomography would be ideal for VAT and SAT quantification, except its application may not be feasible for large populational studies [250, 251].

## Biological mechanisms of obesity in cancer: how fat does what it does

Adipocytes and the adipose tissue are described as dynamic and metabolically active organs, capable of secreting a variety of molecules, with both local and systemic repercussions [252, 253]. Secretion from adipocytes comprehends hormones, cytokines and adipokines, which exert autocrine, paracrine, and endocrine signaling upon many organs [230]. Many mechanisms linking obesity to cancer risk and mortality are suggested, such as insulin resistance, hyperinsulinemia, hyperglycemia, oxidative stress, inflammation and/or adipokine production [254]. Epidemiological evidence about the association of circulating adipokines and cancer include, but are not limited to, breast, gastric, colon, endometrium, kidney, prostate, and pancreatic cancer [240, 246, 255, 256]. In conjunction with obesity-driven chronic inflammation, adipocyte dysfunction plays a fundamental role in adiposity-induced tumorigenesis [31]. This association is clear in cancers developing in or nearby adipocyte-rich environments (particularly breast cancer), and those which invade fat-rich sites, like ovarian and gastric cancers [257–259].

### Adipokines: the communicators of adipose tissue

Adipokines (also called adipocytokines), compose a diverse group of over 20 different hormones and signaling molecules derived from adipocytes [240]. Adipokines are responsible for regulating various physiological processes, including energy balance, lipid metabolism, glucose homeostasis, insulin resistance/sensitivity, angiogenesis, and inflammation. In obesity, the excess of fat tissue results in adipocyte dysfunction and promotes adipose tissue-related disorders (adiposopathies) [254]. Adipocytokines are also implicated in carcinogenesis, tumor progression, recurrence, and metastasis. Between all adipokines, two are of particular importance in obesity and cancer, and will be more thoroughly discussed: leptin and adiponectin. Assuredly, other adipokines have considerable consequences for obesity-related disorders and cancer progression. More comprehensive reviews of the multiple adipokines can be found in the reviews by Saygin et al., and Gallo et al. [240, 260].

### Leptin: a hunger for cancer development

Leptin, the appetite suppressant hormone, was identified in 1994 as the driving factor for obesity in the *ob*/*ob* mice model. This mutation caused early-onset obesity and metabolic alterations, and identification of leptin was essential for consolidating the adipose tissue as an endocrine tissue with effects even upon the brain. The only previously described adipocytokine was a cytokine, TNF-α, in 1993 [261–263]. Leptin has been described as a potent proinflammatory stimulatory hormone on human peripheral blood monocytes [264] and an enhancer of the activation and proliferation of human circulating Th1 lymphocytes [265]. Indeed, impaired cell-mediated immunity is observed in mice with a defect in leptin (ob/ob) or its receptor (db/db) [266–268], and both leptin and leptin receptor are possible targets for intervention in the immunometabolic mediated pathophysiology [269]. Circulating leptin concentrations are closely related to obesity, where higher levels of this hormone are observed in obese individuals [270, 271]. Likely, decreased levels of leptin have been demonstrated in severe malnourished infants [272], whereas an increase in leptin and the immunological recovery was observed after refeeding of malnourished infants [273]. Leptin is classically described as regulator of food intake and energy expenditure, where elevated circulating levels of leptin are associated with adipose tissue inflammation and implicated in breast, colon, prostate, pancreas, ovary, and lung cancers [254, 260, 274–276].

In fact, leptin has a dual role in cancer [277]. Leptin is associated with tumor growth and promotion for its mitogenic, antiapoptotic, pro-angiogenic, and pro-inflammatory effects, while also promoting invasion and migration [240, 254, 278]. The intracellular pathways involved in leptin signaling include JAK/STAT, ERK, and PI3K, culminating in increased proliferation, survival, invasion, and angiogenesis, all of which are hallmarks of cancer development [240]. Leptin can also modulate cell death by inhibiting apoptosis through the upregulation of Bcl expression (namely Bcl-xl, Bak, and Bax) and induce angiogenesis by stimulating HIF-1α and NF-kB, resulting in increased production of vascular endothelial growth factor (VEGF) in breast cancer models. In the MCF-7 cell line, leptin can decrease p53 expression, favoring cancer survival [279, 280]. Mice models of obesity also highlight the role of leptin in survival and maintenance of cancer stem cells, complemented by its regulation of NANOG, SOX2, and OCT4 [281, 282]. Leptin also increases invasiveness, inhibits mitochondrial respiration, and blocks endoplasmic reticulum stress signaling (inhibiting apoptosis) in gastric, colorectal, and liver cancer, respectively [256, 283].

When comparing prostate cancer patients (including advanced stages) to patients with benign prostate hyperplasia or early stage prostate cancer, leptin expression was increased, indicating leptin expression can be used as a biomarker for prostate cancer staging and prognosis [284, 285]. Furthermore, leptin expression is associated to with chemotherapy resistance in gastroesophageal adenocarcinomas, indicating leptin antagonists may be useful in the development of new therapeutic alternatives [286].

### Adiponectin: just like leptin, but you want it

In the context of obesity, another major adipokine is adiponectin, a regulator of glucose homeostasis and fatty acid oxidation, also implied in insulin resistance and diabetes [287]. Rather than leptin, circulating levels of adiponectin are inversely related to adiposity and body fat mass [260, 288]. Obesity, particularly increased visceral fat (also associated with increased oxidative stress and increased inflammation), is accompanied of decreased adiponectin [289]. Accordingly, reduced levels of adiponectin are also a feature observed in different types of cancer and a more aggressive and advanced stage of cancer progression, as observed in breast, colon, esophagus, liver, and endometrial cancer [290, 291]. Indeed, a systematic review and meta-analysis recently showed increased adiponectin was significantly associated with decreased risk of cancer [292]. Adiponectin not only affects cancer cells by directly inhibiting proliferation and invasion, but also indirectly, by reducing insulin resistance and insulin levels [293].

AdipoR1 and AdipoR2 receptors are the best examined receptors that bind to adiponectin. These receptors activate 5′-adenosine monophosphate activated protein kinase (AMPK) and peroxisome proliferator-activated receptor (PPAR)-α pathways, increasing energy expenditure and fatty acid oxidation, while also improving insulin sensitivity [294, 295]. Concurrently, activation of AMPK inhibits the PI3K/Akt/mTOR pathway, increasing expression of p53 and Bax, decreasing DNA replication and translation of cell cycle and angiogenesis genes. This sequence of events causes decreased cell growth and proliferation. Furthermore, low expression of adiponectin receptors (observed in obesity) is associated to endothelial dysfunction, higher histological grade, myometrial invasion, and lymph node metastasis [296, 297]. Taking this into account, adiponectin has opposite effects to leptin in cancer, promoting apoptosis and reducing proliferation, migration, and inflammation [240]. Epidemiological studies agree with the proposed mechanisms, showing low levels of adiponectin are associated to increased number, stage, and risk of endometrial, prostate, esophageal and colorectal cancer [298–301].

### Inflammation: fuel it, and cancer likes it

Given the first described adipokine was TNF-α, a cytokine, the effects of obesity on inflammation, specifically the production of inflammatory cytokines is not an accident. Obesity is extensively described as a state of chronic low-grade systemic inflammation and is an established contributor for cancer progression, fueling inflammation and cancer development by producing inflammatory mediators able to affect the tumor microenvironment [302, 303]. The expansion of adipocytes numbers and area brings with it the apoptosis of these cells. Dying adipocytes are surrounded by monocytes and macrophages, giving rise to crown-like structures, a hallmark of adipose inflammation [304]. The close contact and interaction between adipocytes and immune cells propitiate enhanced production of multiple inflammatory factors, including cytokines, pro-inflammatory adipokines, while also enhancing lipolysis and liberation of FFAs [302, 305]. A growing number of studies has described interactions with adipocytes, such as lymphocytes, eosinophils, neutrophils, mast cells and foam cells [306]. Although there is a lack of knowledge about the initiation of this interaction, macrophage polarization, infiltration of neutrophils, and reduction/dysfunction/exhaustion of T cells are observed and suggested as inducers of adipose inflammation, strengthening the link between obesity and inflammation [307–309].

Alterations in the production and secretion of adipokines can also impact the production of inflammatory factors. Considering the obese phenotype, reduced circulating adiponectin is implied in the production of pro-inflammatory cytokines, such as TNF-α and IL-6, while increased leptin stimulates the production of IL-1, IL-6, IL-12, TNF-α, plasminogen activator inhibitor-1 (PAI-1), leukotriene B4 (LTB_4_) and cyclooxygenase 2 (COX2) [237, 310]. These cytokines are secreted by adipocytes, cancer-associated adipocytes (CAA), macrophages and other immune cells present in the site of inflammation or tumorigenesis [257, 311, 312]. This cytokine cascade creates an ideal tumor microenvironment that facilitates the acquisition of more aggressive and invasive phenotypes [310].

Adipocytes are a major source of TNF-α, secreted in response to FFAs derived from lipolysis (via JNK signaling pathway). TNF-α, on its own, (via ERK signaling pathway) induces lipolysis and, consequently, the release of FFAs. This molecular talk between adipocytes and macrophages gives rise to a positive feedback mechanism, a paracrine loop comprising FFAs and TNF-α that exacerbates inflammation, contributing to a chronic inflammatory dysfunction in the adipose tissue [313, 314]. It is worth to note that TNF-α also contributes to insulin resistance in overall obesity, altering the metabolic landscape [315]. Concurrently, IL-1β and IL-6 expressions are increased. Both cytokines are extensively associated with insulin resistance, tumorigenesis, and cancer progression [316–318]. The presence of adipose tissue and CAAs in the TME (particularly in the fat-rich breast cancer) may aggravate cancer progression and provide the tumor with adipose-derived stem cells, which contribute to cancer development and angiogenesis [302]. Moreover, cancer cells may alter the neighboring adipocytes’ phenotypes, promoting lipolysis, altering the release of adipokines, secreting matrix metalloproteinases, and producing reactive oxygen species in response to FFAs, which, in turn promotes extensive tissue damage and fuels the TME even more [319, 320].

Besides adipokines and inflammation, other processes and pathways are involved in the relationship between obesity and cancer progression. Alterations in insulin-like growth factors’ pathways, induction of hypoxia and HIF-1α signaling, induction of ER stress, alterations in estrogens levels, and modulation of microbiota have all been cited as significant modulators of the effects of obesity in cancer [230, 237, 260].

## Obesity and the obesity paradox in cancer treatment

### Obesity paradox in traditional cancer treatment: the more you try, the less it fits

Obesity and obesity-related disorders have major implications in cancer development and management. Regarding the existence of an obesity paradox, for a few specific types of cancer, obesity can be seen as a phenotypic advantage. Contradicting the evidence of obesity as a risk factor for most cancer types, the obesity paradox has been regarded as an existent, but not largely determinant feature of obesity in cancer. In fact, studies continue to suggest that multimodal interventions, adding body composition, physical fitness, and nutritional and metabolic enhancement of obese individuals to therapeutic intervention, is key for cancer management and treatment [28, 311]. Correspondingly, traditional, well-stablished therapeutic strategies against cancer (chemotherapy, radiotherapy, and surgery) are modulated by the host’s adiposity, with different outcomes and behaviors in the face of treatment. The obesity paradox can be observed in response to cancer treatment, but, as always, evidence is conflicting depending on cancer type, obesity parameters, and treatment [24, 321].

Surgical outcomes in obesity-associated cancer demonstrate both the inexistence of obesity-conferred advantages of high BMI, and the association of high BMI with increased surgical complications in breast, gastric, colorectal, hepatic, and pancreatic cancer [322–325]. Only a handful of cancer types (such as such as gastric, pancreatic and lung cancer) associate obesity to lower mortality and increased long-term survival [326–328]. Notwithstanding, as previously stated, literature discrepancies, methodological biases, and obesity-induced alterations in physiologic states (i.e., cachexia, sarcopenia, insulin resistance/sensitivity) may partly explain these results. Along the same lines, radiotherapy shows inferior outcomes when associated to obesity in breast, prostate, and cervical cancer [329–332].

Chemotherapy, in general, follows the same road, showing reduced efficacy in obese cancer patients, with few available reports linking the obesity paradox to chemotherapy efficiency. On one hand, higher BMI was associated with worse response to doxorubicin and increased hematologic toxicity in breast cancer. On the other hand, obese patients with lung cancer showed no association between treatment with carboplatin and survival/toxicity, indicating obesity may not be detrimental in some types of cancer [28, 333–335]. A recent analysis of patients with lung cancer described decreases in BMI during chemotherapy were associated with poor survival, which would fit the obesity paradox, since higher BMI would be a protective tool [205]. Some studies also show obese patients with less myelosuppression and toxic effects while undergoing chemotherapy, particularly in lung, breast, and gynecologic cancers [336–339]. These findings, however, are debatable since many studies describe obesity as a major factor in promoting cancer resistance to chemotherapy [334, 340–342].

### Poor response to chemotherapy in obesity: just not getting enough?

When reviewing obesity-induced effects on chemotherapy, however, attention must be given to a common practice in the clinic, called dose capping. Dose capping chemotherapy means that obese patients with greater body surface receive lower, or sub-optimal doses of anti-cancer drugs. These inappropriate calculations arise from the use of ideal body weight—instead of the patients’ own body weight—for dose determination [24, 26, 321, 343]. This may be a well-intentioned practice, concerned about excessive toxicity and possible comorbidities in obese patients. However, reduced dosage is associated with reduced survival, and increased cancer recurrence and mortality [344]. Apart from scarce evidence of a positive relation between obesity and chemotherapy efficiency, the underlying biological mechanisms are not yet clear. It was proposed that excessive adipose tissue can dilute drugs, alter blood pressure, and blood flow (affecting drug distribution), and result in liver fat deposition (affecting drug clearance) [28, 336, 345, 346].

When discussing classic, well-established therapeutic strategies against cancer, once again, the obesity paradox is not a rule, but sheds a light upon new mechanistic pathways linking obesity and cancer development. Recent literature still points to one more instance in which obesity seemingly presents beneficial effects: cancer immunotherapy.

## Immunotherapy, cancer, and obesity

### Cancer immunotherapy: stopping cancer’s escape plan

Immunity against cancer involves complex interactions between tumor cells, the host, and the TME, in a process called cancer immunoediting [347]. The understanding of the interplays between the tumor, immune components, and the intrinsic capacity of individuals to fight the tumor are leading to discoveries and adoption of strategies using immunological mechanisms to combat the most diverse types of tumors, as well as the possibility of predicting an individual’s response to immunotherapy [348–350].

Cancer immunotherapy is a therapeutic approach that uses certain components of the body’s immune system to recognize, control and combat cancer cells with greater specificity. There are several strategies that are used to increase immunity against tumor cells, which represent a change in the cancer treatment paradigm [348, 349]. Some of the new immunotherapeutic tools are based on the adoptive transfer of T cells, dendritic cells or Natural Killer (NK) cells, oncolytic viruses, biological modifiers such as vaccines and cytokines, monoclonal antibodies against specific antigens or immunological checkpoint inhibitors, such as inhibitors of cytotoxic T lymphocyte associated protein 4 (CTLA-4), T cell programmed death receptor 1 (PD-1) or one of its ligands (PDL-1 or PDL-2) [350–358].

Many new antigens (neoantigens) associated with tumors can be expressed as mutated proto-oncogenes, expressed in excess, or aberrantly expressed. These so-called tumors associated antigens are antigens that are not naturally expressed in these cell types and are presented via molecules of the major histocompatibility complex (MHC). The recognition of an MHC/peptide complex (pMHC) by a T cell antigen receptor (TCR) is insufficient for complete activation of T lymphocytes. Complete T cell activation requires a second co-stimulatory signal, in which receptors and ligands on the surface of professional antigen presenting cells (APC) stimulate T lymphocyte activation, differentiation, proliferation and function. There are several receptor/ligand combinations between T cells and APCs which can induce stimulatory signals, such as the coupling between CD28 and CD80/86, or inhibitory signals, such as PD-1 and PD-L1/PD-L2. These inhibitory signals are called immune checkpoints [349].

Immune checkpoint molecules, like CTLA-4, PD-1, lymphocyte-activation gene 3 (LAG3), T-cell immunoglobulin and mucin-domain containg-3 (TIM-3), and V-domain Ig suppressor of T cell activation (VISTA), among others, are important negative regulators of immune system activation and are generally expressed by APCs and regulatory T cells to inhibit cytotoxic T cells and preserve tolerance [359]. The inhibition of T cells leads to the suppression of the activation of these cells, inducing a state of anergy, with decreased proliferation, effector functions, and production of cytokines. The checkpoint molecules can also be expressed in high and sustained levels by CD4+ or CD8+ T cells activated in response to factors derived from the TME, indicating a state of cellular exhaustion. The ligands for these inhibitory receptors can be found in tumor cells and several myeloid cells [359–361]. Cancer cells are capable of molding the TME, inhibiting T cells by engaging with immunological checkpoints, and recruiting non-tumor cells such as fibroblasts, macrophages, and endothelial cells, amongst others. These components of the TME help and protect tumors from immune system recognition and elimination, and, thus, circumvent the tumor immunovigilance (or immunosurveillance), resulting in the escape of tumor cells from the immune system response [347, 349, 359, 362].

Tumor cells, however, can also develop a different interrelation with the immune system. Immunoselective pressure drives a process called immunoediting, in which tumor cells shape the antigenic repertoire of their subclones, eliminating the more immunogenic variants. Tumor cells can further subvert infiltrating immune cells, using them to promote their growth, tumor progression and inducing an immunosuppressive microenvironment [347, 363, 364]. Polarization of immune cells leading to a pro-inflammatory cytokine profile, or infiltration of innate and adaptive immune cells in the tumor may contribute to tumor progression, instead of killing. Reportedly, macrophages, neutrophils, B cells, immature and mature dendritic cells, regulatory T cells, natural killer T cells, and increased ratios of CD4/CD8 or Th2/Th1 T cells have been described as participants of this process [363–365].

Antibodies that inhibit immunological checkpoint molecules were conceived based on the hypothesis that blocking immune inhibitory signals could restore effector activity of T cells or boost T cell priming. Accordingly, checkpoint inhibitors interrupt the inhibition signaling of checkpoint molecules, promoting antitumor effector responses. With preclinical and clinical advances, antibodies such as ipilimumab (anti-CTLA-4 antibody), nivolumab, pembrolizumab (anti-PD-1 antibodies), durvalumab and avelumab (anti-PD-L1 antibodies) have been approved by the United States’ Food and Drug Administration (FDA). Several other antibodies targeting other immunological checkpoint molecules are under development and have shown promising results against several types of cancer [366]. In the last decade, immunotherapy revolutionized cancer therapy and is currently a centerpiece of adjuvant and neoadjuvant therapies, by itself or combined to chemotherapy and/or radiotherapy [367, 368].

### Immunotherapy response-determinant host factors: you change, immunotherapy changes

In addition to understanding the TME, recent studies have also demonstrated the importance of patient-associated factors that can impact the response to immunotherapy, such as body composition (fat mass, fat-free mass and muscle quality), sex, ethnicity, age, habits (e.g., smoking, alcohol use, physical activity), past illnesses (immune history) and gut microbiota [23, 24, 369–373]. Obesity, currently considered a pandemic, is characterized by changes in the physiology of the entire body. Compromised body homeostasis due to obesity is associated with unregulated immune responses and chronic systemic inflammation, involving inflammatory factors such as leptin [374–377]. Paradoxically, these changes can be associated either with the appearance and progress of malignancies or as a protective phenomenon in certain types of tumors. These changes can be understood as the appearance of tumor cell phenotypes that can be more or less favored in disturbed systems, such as in patients with obesity [375, 378, 379]. Recent studies have mainly demonstrated the correlation between obesity and the response rate to immunotherapies of immune checkpoint inhibitors [199, 377, 380].

### Obesity as a factor in immunotherapy success: hitting the checkpoint

Obesity impacts several features of T cell responses, aiding the immune escape of tumors through many mechanisms. It has been shown that in obesity, there is a decrease in the proliferative capacity of T cells, production of interferon gamma (IFNγ) and TNF-α, and increased expression of PD-1, which may be associated with leptin signaling [377]. Signaling promoted by leptins leads to increased pro-inflammatory T-helper 1 (Th1) immune responses, and its relationship with increased exhaustion and T cell dysfunction in obese patients is probably due to a phospho-signal mediated by the transducer and activation of transcription 3 (STAT3) pathway [268, 376, 381, 382]. Wang et al. demonstrated that, in obese animals, changes in PD-1 expression may contribute to a higher response rate to immune checkpoint blockade (ICB) therapy with PD-1/PD-L1 inhibitors, leading to increased infiltration of CD8+ T cells in subcutaneous tumors, reduction in the number of lung metastases and increased overall and progression-free survival. In human colorectal cancers, a low response to immunotherapy with anti-PD-1/PD-L1 is generally observed, and it has been shown that there is a smaller number of T cells in the tumor of obese compared to non-obese patients. In another cohort of patients with melanoma, a tumor with favorable of response to ICBs, there was a significant increase in the expression of PD-1 in tumors of obese patients. In obese animals, increased tumor progression has also been demonstrated, probably due to the induction of an immunosuppressive state and effects mediated by metabolites and hormones, such as leptin [377]. The chronic inflammation process, frequently observed in obese patients, can also induce immunosuppression, a protection mechanism against possible autoreactive responses of the immune system.

## Immunotherapy and the obesity paradox

### Obesity paradox in immunotherapy: a place to call home?

When trying to establish hypothesis and cause-consequence effects of the obesity paradox, an obstacle is the fact that obesity itself is multi-faceted and induces macro, systemic physiological alterations, making it difficult to determine beginning and endpoints for the observed associations with cancer development and treatment. By focusing observations of the obesity paradox on more micro, specific pathways, mechanisms can be more thoroughly analyzed.

It has been well established that obesity can trigger severe modulation of the immune landscape, resulting in metabolic and immunological dysfunction. In this way, adipose tissue from individuals with obesity can display immunological and metabolic modifications that can deeply impact cancer developing, as well as antitumor immunotherapies, since the chronic low inflammatory state associated with obesity has varied effects on anti-cancer immunity and immunotherapy effectiveness. ﻿Indeed, adipose tissues can be highly plastic and strongly impact inflammatory response and cancer progression by several different mechanisms [18]. Obesity can accelerate thymic aging, compromising T lymphocytes formation and proliferation, and consequently impairing progenitor pool and generation of the restricting the T cell repertoire [383]. Moreover, people with obesity can often display T lymphocytes with an exhausted phenotype in phenomenon that can be orchestrated by adipocytes and macrophages as a result of chronic inflammation due to prolonged stimulation of toll-like receptors by circulating free fatty acids, adipocyte cell death, activated stress responses, and hypoxia [384].

**Fig. 3:**
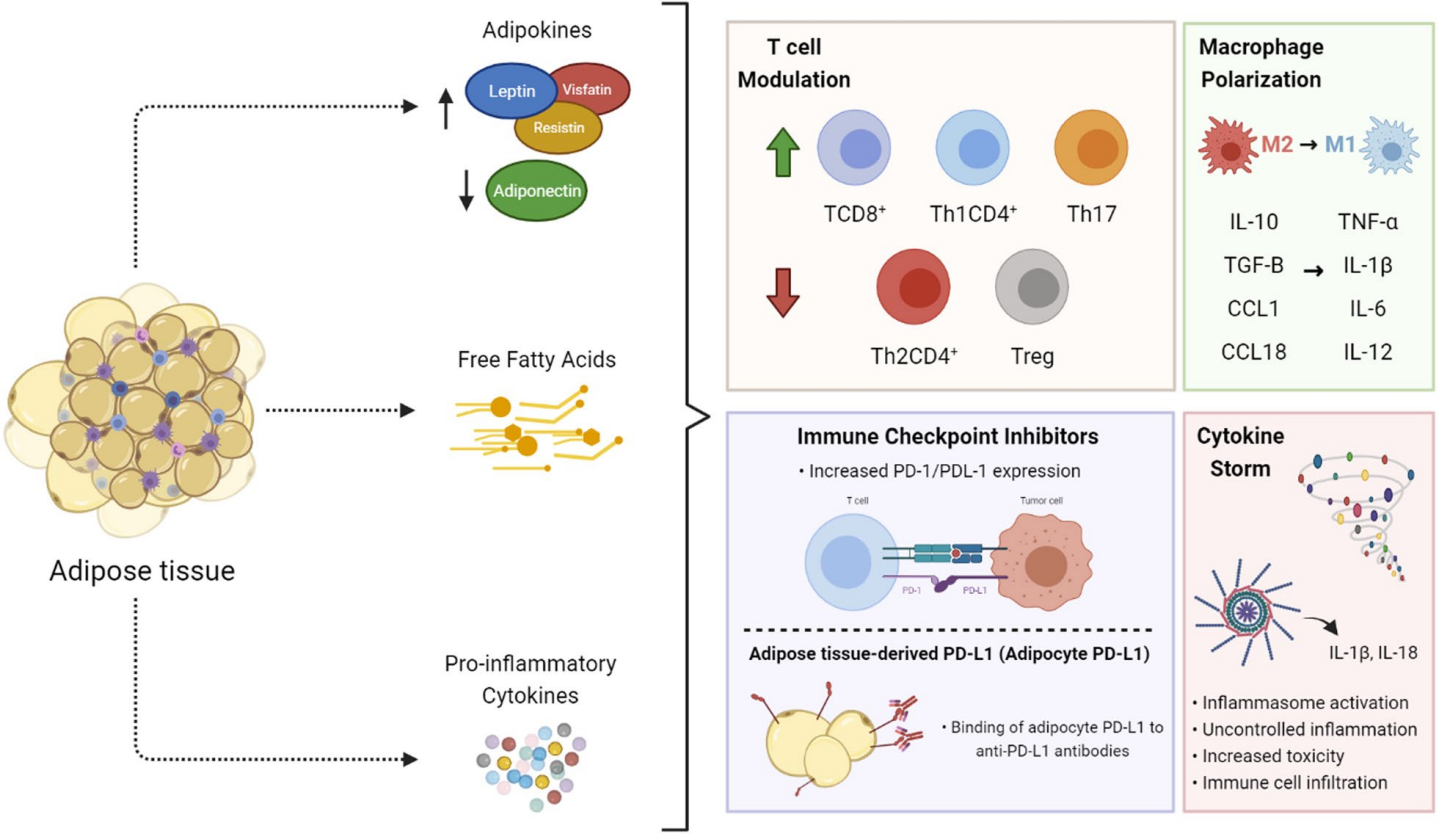
Implications of obese adipose tissue upon the immune system and immune checkpoint proteins. The dysregulation of the secretion of adipokines, free fatty acids and pro-inflammatory cytokines by the adipose tissue from individuals with obesity has a number of implications upon the immune system. These factors regulate both the proliferation of T cells, as well as their exhaustion, via modulation of PD-1 and PD-L1 expression, also affecting the antitumor immune response against cancer and immune checkpoint inhibitor immunotherapy. The polarization of macrophages from a M2 to a M1 profile induces changes in inflammatory cytokines production, often resulting in a ‘cytokine storm’, an event of uncontrolled inflammation which results in toxicity and failure of different organs

At first, obesity was thought to increase toxicity and impair immune efficacy, which makes sense with observations of T-cell inflammaging-like processes in a many animal species and, particularly, humans [377]. It has been showed that obesity induces T cell dysfunction and an upregulation of PD-1 on T lymphocytes, in a partially leptin-dependent manner. However, the polarization of T cells towards an exhaustive phenotype is correlated with improved response rates to anti-PD-1 therapy in the setting of obesity. Indeed, recent studies comparing obese to lean patients undergoing immunotherapeutic treatment show improved response to immunotherapy, together with longer progression-free and overall survival [385]. These obesity-favored effects were specially observed in checkpoint blockade targeting PD-1/PD-L1 of patients with multiple cancer types [199, 377] (Fig. 3). Therefore, obesity, which is associated with T cell dysfunction and worsen cancer prognostic, also paradoxically induces a better response to anti-PD-1/PD-L1 immunotherapy [386–388].

In the context of this obesity paradox, different mice models resulted from genetic ablation or diets can be used to understand better the role of obesity in cancer progression and anti-tumor immunotherapy during preclinical studies. Diet-induced obesity animal models can be frequently established by feeding animals with different diets containing for instance a high proportion of sugar (high-sugar diet) or fat (high-fat diet), or a combination of high-fat and high-carbohydrates in a diet called, cafeteria diet [389–391]. Amongst genetic animal models, mice with deficient leptin signaling are the most regularly used [262]. In this model, obesity can be induced by a mutation in the leptin receptor gene (db/db mouse) or by the lack of leptin (the ob/ob mouse), both causing the mice to overfeed.

Murphy and colleagues identified leptin as a potential therapeutic target for neutralization to enhance immunotherapy efficacy in obese cancer patients [392]. By using a leptin-deficient (ob/ob) obese BALB/c mice fed a high-fat diet model these authors demonstrated that systemic anti–CTLA-4 mAb and intratumoral delivery of a TRAIL-encoding adenovirus plus CpG immunnotherapies were effective in lean mice, but not in diet-induced obese BALB/c mice [392]. These data highlighted the potential of targeting leptin to boost cancer immunotherapy when in individuals with obesity.

Genetic mice models which allow a rapid development of highly metastatic tumors can be also used to study the obesity paradox in cancer development. Using a MMTV-PyMT mice as breast cancer model, Cranford and colleagues showed that high fat diet-induced obesity lead to an increased hormone production via aromatase expression and inflammation, enhancing breast cancer tumorigenesis [393]. Moreover, the MMTV-PyMT mice model has been used to test CTLA-4 or PD-1 checkpoint inhibition effects [394]. Additionally, Gibson and colleagues also investigated the effect of obesity on MDSC-mediated immunotherapy resistance in a breast cancer mouse tumor model, showing that obesity could trigger the accumulation of FasL+ granulocytic MDSCs, inducing apoptosis of tumor-infiltrating CD8 T lymphocytes and immunotherapy resistance in this cancer [395].

There is complex balance among diet, gut microbiota diversity, obesity, and the regulation of immune and inflammatory responses which can directly impact cancer development and antitumor immunotherapy. Obesity, gut microbiota and inflammatory signaling pathways are deeply connected. It has been showed that mice fed a high-fat diet alters gut microbiota [396] and mice lacking inflammasome’s caspases 1/11, but not NLRP3, were more susceptible to high fat diet-induced weight gain and presented higher abundance of phyla related to inflammation and gut dysbiosis, compared to wild type mice [397]. In another study, gut dysbiosis and high-fat diet present in a mice with predisposition to develop intestinal cancer (Kras^G12Dint^) accelerated the development of intestinal tumors [398]. In fact, gut microbiota function as strong regulator of antitumor immune response. Administration of antibiotics and fecal microbiota transplantation might impact the efficacy and toxicity of immunotherapy via gut microbiota [399–401].

### Obesity paradox in melanoma immunotherapy: right under my skin

Currently, melanoma is highly targeted by immunotherapies, with seven different FDA-approved options for treating this type of cancer. These include oncolytic virus therapy and immune modulators, with marked observations using immune checkpoint inhibitors, such as ipilimumab, pembrolizumab, and nivolumab (targeting CTLA-4 and PD-1), or a combination of these [402, 403]. Notably, high BMI immunotherapy-treated melanoma patients show favorable outcomes, where several clinical studies report favorable associations between high BMI and immune checkpoint therapy in the context of melanoma, with or without accompanying chemotherapy [385, 404]. In these situations, overweight melanoma patients, when compared to lean patients, show better progression-free and overall survival. Of note, there is no difference in observations using PD-1 or PD-L1 immunotherapy [199, 377, 387, 402, 405].

In animal models of obese melanoma-bearing mice, anti-PD-1 immunotherapy enhances response to cancer by increasing number and function of tumor-associated CD8+ T cells, alongside a decrease in PD-1 expression by T cells, implying that the blockade of PD-1 is able to overcome obesity-driven T-cell exhaustion [377]. Leptin has also been associated with PD-1 expression and T-cell exhaustion. Leptin-induced TME immunosuppression is mediated by increased PD-1 expression and upregulation of activated STAT3, a mediator of leptin signaling which interacts with the PD-1 gene promoter [406]. On the other hand, in mice models of obesity, there was no improvement of anti-CTLA-4 treatment response until leptin was neutralized using soluble mouse leptin receptors, which lead to an increase in co-stimulatory CD86 expression [392]. This contradictory effect of leptin depending on the target of immunotherapy reveals the complexity of leptin signaling in innate and adaptive immune responses [407]. Obesity-induced alterations in gut microbiota have also been linked with the efficacy of ICB [372, 408].

Another important factor in determining favorable response to anti-melanoma immunotherapy is sex-related differences in hormone production, mainly estrogen, already implicated in the regulation of innate and adaptive immunity [409]. Indeed, female melanoma patients outperform males when comparing their response to immunotherapy [410, 411]. Even though melanoma does not express classical estrogen receptors, it does express G-protein-coupled estrogen receptors (GPERs), which, when activated, decrease PD-L1 expression and increase tumor susceptibility to T cells [412]. Studies regarding anti-PD-1 immunotherapy in mice showed synergy between ICB and GPERs activation, resulting in tumor regression, extended survival, and improved immune memory [412, 413].

### Obesity paradox in NSCLC and renal cell carcinoma immunotherapy: it fits

A common trend of the obesity paradox in cancer is being true in a few specific cases but failing to address the benefits of obesity in the majority of cancer types. Since data availability of the correlation between BMI and immunotherapy efficacy is still very much scarce, no definitive conclusion about the extent of obesity paradox in immunotherapy can be given. Nonetheless, recent literature does reflect its existence and benefits in non-melanoma cancer, such as NSCLC, and renal cell carcinoma.

Throughout this review, evidence already pointed to the benefits of obesity in NSCLC when analyzing other parameters than response to immunotherapy. Although descriptions of the obesity paradox in lung cancer are not so mechanistically profound, epidemiological evidence is plenty. At least five different studies report a positive association between obesity (especially subcutaneous fat mass) and improved outcomes in patients receiving immune checkpoint inhibitors [414]. When compared to lean individuals, obese patients treated with pembrolizumab, nivolumab, or atezolizumab showed better and improved response to treatment, prolonged progression-free survival, and increased overall survival. These results are also true for advanced stages of lung cancer [415–418]. Together, they point to the existence of the obesity paradox in lung cancer immunotherapy, similarly to melanoma. Of note, Magri et al. used computerized tomography to analyze their data, which confirmed results had with BMI alone [419]. Curiously, no correlation between BMI and improved outcomes were observed for patients treated with chemotherapy, suggesting that, in lung cancer, at least, the clinical benefits of the obesity paradox are unique to immunotherapy [415].

Renal cell carcinoma (RCC) is another cancer subset reported to fit in the obesity paradox of cancer immunotherapy [420]. Three different studies comparing obese and lean patients treated with nivolumab or ipilimumab showed BMI had positive association with overall progression-free and overall survival, even when no correlation between BMI overall response rate, and disease control rate was found [421–423]. Conflicting reports, however, do exist. A study by Boi et al. associated obesity to worst overall and progression-free survival in RCC patients receiving standard anti-PD-1 immunotherapy [424].

### Obesity paradox in breast cancer immunotherapy: the host’s contribution

The efficiency of anti-PD-1 immunotherapy for breast cancer (particularly TNBC) is sparse [425]. In fact, one of the few reports in which obesity is associated with better progression-free and overall survival of breast cancer patients reports the use of different immunotherapeutics, in this case, bevacizumab, an inhibitor of VEGF-A [260].

An obstacle for the success of anti-PD-1 immunotherapies in breast cancer is the recently described production of PD-L1 by the host’s adipose tissue [425]. This marked feature of breast cancer patients adds to the complexity of the obesity paradox in cancer immunotherapy. Breast cancer TME is an adipocyte-rich environment, therefore, secretion from adipocytes is particularly important. Proposed mechanisms for the interference of adipose PD-L1 with anti-PD-1/PD-L1 immunotherapy include binding of adipocyte PD-L1 to anti-PD-L1 antibodies, and interaction of adipocyte PD-L1 with T-cell PD-1 directly. Accordingly, knockout models for adipocyte PD-L1 alter T cell activation and tumor killing [426, 427]. Yet, the FDA recently approved the use of anti-PD-L1 atezolizumab for TNBC, and anti-CTLA-4 immunotherapies are currently under clinical trials [428].

### Immune-related adverse events: immunotherapy success is not for free

Overall, immunotherapy is a successful, promising, and more personalized treatment for cancer patients. Regardless of its efficacy, immunotherapy, by its own purpose and immunosuppressive action mechanisms, can induce adverse events, denominated immune-related adverse events (irAEs). The immune checkpoints upon which immunotherapy exerts its effects are spread over many different immune cells (Treg cells, MDSCs, γδ T cells, TAMs, etc.), and their blockage alters the immune homeostasis, possibly leading to a series of autoimmune incidents [429]. Unbalance of T effector/T regulatory cells ratios and overactivation of T lymphocytes (including cytotoxic T cells) may provoke killing of normal, non-transformed cells, induce the release of neoantigens, tumor antigens and auto-antigens, and result in the release of large amounts of pro-inflammatory cytokines (IFN-γ, IL-17 and IL-6, from Th1 and Th17 T lymphocytes), culminating in a lethal cytokine storm [430–433].

Obesity-associated inflammation can also impact the immune response and consequently affect the efficacy and toxicity of immunotherapy. Apart from the beneficial aspects of obesity in cancer immunotherapy, the persistence of inflammation in obese patients has been linked to the development of irAEs [30]. Indeed, some studies associated higher BMI with increased risks of irAEs after immunotherapy [414, 434, 435]. In addition, limited evidence is available on the efficacy of immunotherapy upon obese and lean patients, which draws attention for considering BMI when designing randomized clinical trials for newer therapies [321].

## Conclusion

The established link between obesity and several types of cancers raise important questions regarding conversion of this knowledge into actual and effective protective measures against cancer. Undoubtedly, maintenance of a healthy weight is an evident stage to potentially reducing risk of obesity-related cancers. However, even though elevated BMI is associated with increased cancer incidence for several cancer types, a number of studies have emerged evidence that cancer patients with obesity can present an improved survival rate upon immunotherapy against cancer. Although, this therapeutic advantage of a higher BMI is observed in few cancers during anti-tumor immunotherapy, millions of patients with obesity and cancer could benefit from this increasing evidence about the underlying physiological and biological mechanisms of obesity paradox’s effects upon cancer treatment and survival.

## Data Availability

Not applicable.

## Abbreviations

(AMP): Adenosine monophosphate
AGE: Advanced glycation end product
AIM2: Absent in melanoma 2
*AMPK*: Activate 5′-adenosine monophosphate activated protein kinase
APC: Antigen presenting cells
AT: Adipose tissue
ATM: Tissue microenvironment
ATP: Adenosine triphosphate
BAT: Brown adipose tissues
BMI: Body Mass Index
CAA: Cancer-associated adipocytes
CD: Cluster of differentiation
CLS: Crown-like structures
COX2: Cyclooxygenase 2
CTLA-4: Cytotoxic T lymphocyte associated protein 4
CVD: Cardiovascular disease
DAMP: Damage-associated molecular patterns
ER: Endoplasmic reticulum
ER: Estrogen receptor
ERK: Extracellular signal-regulated kinases
ETC: Electron transport chain
FDA: Food and Drug Administration
FFA: Free fatty acids
GPERs: G-protein-coupled estrogen receptors
GSDMD: Gasdermin D
HFD: High-fat diet
HIF-1a: Hypoxia-inducible factor 1-alpha
HMGB1: High Mobility Group Box-1
ICB: Immune checkpoint blockade
IFNγ: Interferon-gamma
IL: Interleukin
ILC: Lymphoid cells
IR: Insulin resistance
irAE: Immune-related adverse event
JAK: Janus kinase
JNK: C-Jun N-terminal kinase
LAG3: Lymphocyte-activation gene 3
LPS: Lipopolysaccharide
LTB_4_: Leukotriene B_4_
LTB_4_: Leukotriene B4
MCP1: Monocyte chemoattractant protein-1
MDSC: Myeloid-derived suppressor cells
MHNO: Metabolically healthy non-obese
MHO: Metabolically healthy obese
MMTV-PyMT: Mouse mammary tumor virus-polyoma middle tumor-antigen
MS: Metabolic syndrome
mTOR: (Mammalian target of rapamycin)
MUNO: Metabolically unhealthy non-obese individual
MUO: Unhealthy obese individual
NAFLD: Non-alcoholic fatty liver diseases
NF-κB: Nuclear factor kappa-light chain-enhancer of activated B cells
NK: Natural killer
NLR: NOD-like receptors
NLRP3: NOD-like receptor protein-3
NOD: Nucleotide-binding oligomerization domain
NSCLC: Non-small cell lung cancer
OCT4: Octamer-binding transcription factor 4
PAI-1: Plasminogen activator inhibitor-1
PD-1: Programmed death receptor 1
PDL-1: Programmed death receptor ligand 1
PI3K: Phosphoinositide 3-kinase
RAGE: Receptor for advanced glycation end product
RCC: Renal cell carcinoma
ROS: Reactive oxygen species
SAT: Subcutaneous adipose tissue
SOX2: Sex determining region Y-box 2
STAT: Signal transducer and activator of transcription proteins
T2D: Type 2 diabetes
TAM: Tumor associated macrophages
TCR: T cell antigen receptor
Th: Helper T lymphocytes
TIM-3: T-cell immunoglobulin and mucin-domain containg-3
TLR: Toll-like receptors
TME: Tumor microenvironment
TNBC: Triple-negative breast cancer
TNF-α: Tumor necrosis factor-alpha
TRAIL: Tumor necrosis factor-related apoptosis-inducing ligand
Treg: Regulatory T lymphocytes
VAT: Visceral adipose tissue
VAT: Visceral adipose tissue
VEGF: Vascular endothelial growth factor
VISTA: V-domain Ig suppressor of T cell activation
WAT: White adipose tissue
